# Microfluidic technologies for immunotherapy studies on solid tumours

**DOI:** 10.1039/d0lc01305f

**Published:** 2021-06-04

**Authors:** K. Paterson, S. Zanivan, R. Glasspool, S. B. Coffelt, M. Zagnoni

**Affiliations:** Centre for Microsystems and Photonics, EEE Department, University of Strathclyde Glasgow UK michele.zagnoni@strath.ac.uk; Institute of Cancer Sciences, University of Glasgow Glasgow UK; Cancer Research UK Beatson Institute Glasgow UK; Beatson West of Scotland Cancer Centre Glasgow UK

## Abstract

Immunotherapy is a powerful and targeted cancer treatment that exploits the body's immune system to attack and eliminate cancerous cells. This form of therapy presents the possibility of long-term control and prevention of recurrence due to the memory capabilities of the immune system. Various immunotherapies are successful in treating haematological malignancies and have dramatically improved outcomes in melanoma. However, tackling other solid tumours is more challenging, mostly because of the immunosuppressive tumour microenvironment (TME). Current *in vitro* models based on traditional 2D cell monolayers and animal models, such as patient-derived xenografts, have limitations in their ability to mimic the complexity of the human TME. As a result, they have inadequate translational value and can be poorly predictive of clinical outcome. Thus, there is a need for robust *in vitro* preclinical tools that more faithfully recapitulate human solid tumours to test novel immunotherapies. Microfluidics and lab-on-a-chip technologies offer opportunities, especially when performing mechanistic studies, to understand the role of the TME in immunotherapy, and to expand the experimental throughput when using patient-derived tissue through its miniaturization capabilities. This review first introduces the basic concepts of immunotherapy, presents the current preclinical approaches used in immuno-oncology for solid tumours and then discusses the underlying challenges. We provide a rationale for using microfluidic-based approaches, highlighting the most recent microfluidic technologies and methodologies that have been used for studying cancer–immune cell interactions and testing the efficacy of immunotherapies in solid tumours. Ultimately, we discuss achievements and limitations of the technology, commenting on potential directions for incorporating microfluidic technologies in future immunotherapy studies.

## Abstract 2

Immunotherapy is a powerful and targeted cancer treatment that exploits the body's immune system to attack and eliminate cancerous cells.

## Graphical abstract

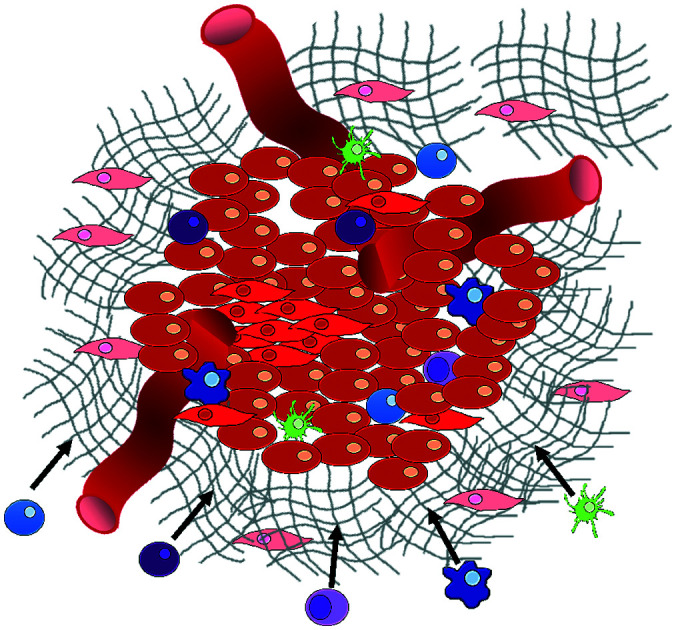

## 1. Introduction

Immuno-oncology (I/O) is defined as the study and development of therapies that exploit the immune system to fight cancer.^1^ Immunotherapy has the potential to harness the intrinsic capabilities of the innate and adaptive immune system to identify, target and eradicate cancer cells regardless of the tissue they affect. In contrast to conventional anti-cancer therapies, which do not distinguish between healthy and cancerous cells, immunotherapy can sometimes offer specific cancer cell killing and prevention against recurrence due to the memory capabilities of the immune system.^2^ However, only a fraction of cancer patients benefit from the current repertoire of immunotherapies.^3^ As a result, researchers are left with the challenge of enhancing the effectiveness of existing immunotherapies, identifying predictive markers, and discovering new immune pathways for intervention. For these endeavours to succeed, better preclinical model systems that can guide personalized immunotherapy treatments are required.

Solid tumours and their microenvironment, including hypoxic conditions, acidic pH, nutrient starvation, dysfunctional vasculature and the occurrence of immunosuppressive mechanisms, present significant challenges for immune cells. Microfluidic technologies can recapitulate many of these environmental features, offering a versatile tool to study solid tumour immunotherapy and representing valuable pre-clinical modelling systems.^4^ By allowing greater control of fluid volumes, culture conditions, surface chemistry and stimuli exposure, the unique characteristics of the technology make microfluidics an ideal platform for the development and testing of immunotherapeutic agents.^5–7^ Microfluidic technologies are often used to test anti-cancer therapies on liquid,^8,9^ and solid tumours, such as spheroids, organoids^10^ and tumour tissue slices.^11^ They are also used to study immune cell behaviour, interaction and migration in a reconstructed tumour model, but have not been extensively used for efficacy studies of cancer immunotherapy. Thus, microfluidic technologies offer an underutilized resource for I/O.

In this review, we discuss the achievements to date made by using microfluidic platforms for I/O investigations that could not have been achieved *in vivo* or with standard *in vitro* off-chip techniques. We first present an overview of the present immunotherapeutic strategies for treating solid tumours and their clinical limitations. Finally, we present an outlook on future opportunities and challenges for microfluidics in the I/O field that could impact the progression of immunotherapy research.

## 2. Immunotherapies for solid tumours

**Fig. 1:**
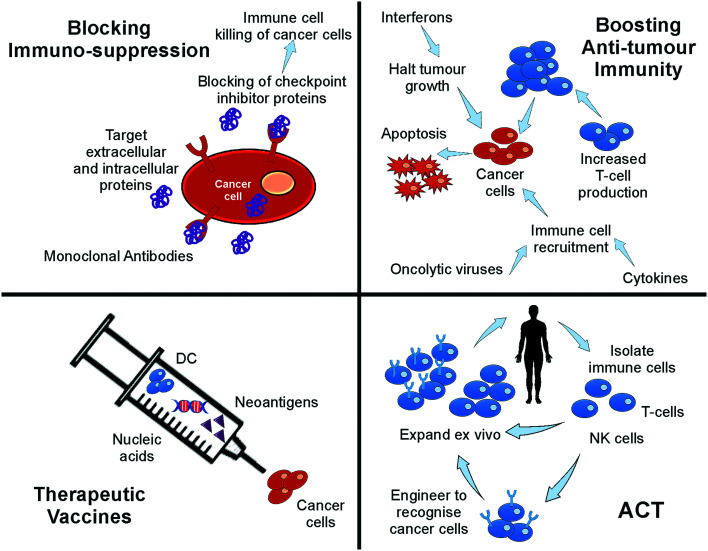
Immunotherapy strategies. Schematic drawing of the most common immunotherapeutic methods and their mechanism of action (dendritic cells (DC), adoptive cell transfer, (ACT)).

Common immunotherapeutic approaches (Fig. 1) and their mode of action are described here briefly, directing the reader to field-specific reviews, prior to discussing their implementation for I/O in solid tumours using microfluidic technologies.

*Immune checkpoint inhibitors* (ICIs) are the most successful type of cancer immunotherapy to date. Immune checkpoint molecules (or co-inhibitory molecules as they are also called) are an inherent, natural system to regulate the magnitude of immune response.^12^ ICIs prevent the interaction between checkpoint molecules expressed on T cells and their ligands expressed on antigen-presenting cells or cancer cells. This inhibition unleashes greater T cell killing of cancer cells. Two of the most well-studied checkpoint molecules include cytotoxic T-lymphocyte-associated antigen 4 (CTLA-4) and programmed cell death protein 1 (PD-1).^13^ Ipilimumab, which targets CTLA-4, was the first ICI licensed for use in cancer patients, in the treatment of metastatic melanoma.^14^ The first two PD-1 ICIs to be approved were pembrolizumab and nivolumab, which show similar efficacy and longstanding results as ipilimumab. These drugs are effective in more than 25 types of solid cancers and multiple liquid malignancies.^14,15^ However, response rates to anti-CTLA-4 and anti-PD-1 therapies vary by cancer type with upper limits reaching only 40% in melanoma and lung cancer. Further improvements to ICIs could benefit a great number of cancer patients.

*Adoptive cell transfer* (ACT) therapy involves the administration of immune cells to a patient. These cells can be allogeneic (taken from a donor) or autologous cells (taken from the patients' own immune cells) that have been isolated and subsequently modified and expanded *ex vivo*, before being returned to the patient.^16,17^ ACT products include tumour-infiltrating T cells, T cell receptor (TCR)-transduced T cells, chimeric antigen receptor (CAR)-T cells, NK cells and CAR-NK cells. Thus far, ACT products have had success in treating melanoma, head & neck cancer, renal cell carcinoma, gynaecological cancers as well as leukaemia and lymphomas.^14^ NK cell-based immunotherapy has been mostly investigated for haematological malignancies, such as acute myeloid leukemia (AML) and acute lymphoblastic leukaemia (ALL). It has shown limited success in multiple solid malignancies due to a lack of tumour infiltration and proliferation.^14^ In addition to the immunosuppressive tumour microenvironment limiting immune cell infiltration, acquired mutations by cancer cells may provide additional escape mechanisms to prevent T cell and NK cell attack.^18^ CAR-T cells are engineered to express a receptor specific to an antigen on the surface of cancer cells, such as CD19. These cells are used in leukemia and lymphoma patients,^17^ and they can stay active for up to a decade once injected, potentially being a one-time therapy.^13^ Tisagenlecleucel and axicabtagene-ciloleucel were the first to be approved for all and certain types of large B-cell lymphoma.^15^ For solid tumours, CAR-T cells are still being trialled in such cancers as lung, brain, breast, pancreatic, sarcoma and metastatic colon cancer.^14^ However, a lack of suitable cancer-specific antigens is a major limitation in the CAR-T cell field.^3,15^ Other challenges for CAR-T therapy include the laborious, expensive and time-consuming production.^13^ In solid tumours, CAR-T can be ineffective and fail to persist; although, there are examples of progress in this area, such as CAR-T cells targeting EGFR-expressing glioblastoma.^13^

*Therapeutic vaccines* consist of recombinant viral, bacterial and yeast vectors, immunogenic peptides, immune cells or killed tumour cells, which are used to improve immune system activation against cancer cells.^13,19–22^ Cancer vaccines stimulate dendritic cell function resulting in greater T cell responses. This said, there has been little clinical success for cancer vaccines thus far when administered alone. However, promising results have been obtained when used in combination with checkpoint inhibition.^14^ The Bacillus Calmette–Guérin (BCG) vaccine is a nonspecific immune stimulant that was the first immunotherapy to receive FDA approval in 1990 to treat bladder cancer and remains the sole intravesical agent to prevent non-muscle invasive bladder cancer (NMIBC) progression.^23^ Neoantigen vaccines have shown anti-tumour benefit for melanoma and glioblastoma, whilst the TG01 mutant K-Ras peptide vaccine has been approved for pancreatic cancer.^14^ Dendritic cell vaccines are the most investigated vaccine type with sipuleucel-T approved in 2010 for improving the overall survival in prostate cancer patients, despite its complex production and moderate efficacy.^13,19^ Thus, there is room for great improvement in cancer vaccine technology.

Another type of immunotherapy involves *oncolytic viruses*, which use the innate ability of a virus to kill cancer cells with the potential to initiate an anti-tumour response. Oncolytic viruses reproduce inside and destroy cancer cells, whilst leaving normal cells unharmed and triggering the recognition of antigens released from the lysed cancer cells that induces further anti-tumour immune responses.^14^ Oncolytic viruses can be beneficial in treating melanoma and brain cancer.^15,18^ With regard to solid tumours, intratumoural injection are ideal for direct cancer cell lysis without detrimental systemic effects or hepatic virus degradation.^18^ Unfortunately, this procedure is not technically possible for most metastatic cancers. Roadblocks for this form of therapy, in addition to those caused by the tumour microenvironment (TME) and in particular the dense fibrotic tissue surrounding tumours, include complete viral clearance, and acquired specific immunity against the virus, meaning that repeat therapy is not possible.^14,18^

To stimulate anti-tumour immunity in cancer patients, *cytokine therapy* may be employed. Cytokines, such as interferons, interleukins, and granulocyte-macrophage colony-stimulating factor (GM-CSF), can boost T cell responses, activate apoptosis programs in cancer cells, delay angiogenesis, stimulate dendritic cell maturation, and slow cancer progression.^13,18,24^ A disadvantage to the use of cytokines, however, is their short half-life. This means that treatment needs to be delivered at a high dose, which can result in serious side effects, such as cytokine release syndrome (CRS), vascular leak syndrome and autoimmune attacks.^13^

For many immunotherapies to be successful, immunosuppression must be overcome and anti-tumour immunity must be engaged. This may be accomplished by targeting immunosuppressive enzymes, such as indoleamine-2,3-dioxygenase (IDO1) and arginase.^25^ The STING pathway, which is a cytoplasmic DNA-sensing system (either self DNA or non-self DNA such as viral DNA), represents another area of active research for immunotherapy, because it activates anti-cancer immune responses. The STING agonist, ADU-S100, benefits patients with triple negative breast cancer and melanoma when given in combination with spartalizumab, a checkpoint inhibitor.^26^

Targeting solid tumours with immunotherapies is a field in development. Many 2D and 3D *in vitro* models have been established to investigate the effects of therapeutic agents,^27^ with reports of immune cells being unable to kill cancer cells in a 3D environment, despite doing so in 2D assays,^28^ or with anti-tumour effects of T cells being significantly reduced in 3D cultures.^29^ The complexity originating from the intricate spatial cellular organizations within the TME and their interactions with immune inhibitory mechanisms highlights the need for complex *in vitro* models that faithfully mimic human *in vivo* scenarios.^30,31^ Various solutions to these challenges can be provided by microfluidic and organ-on-a-chip technologies that enable miniaturisation, maximise the use of intact human tumour tissue, allow the robust and controlled modelling of tumour microenvironment characteristics and facilitate the culture of multiple cell types in defined spatial and temporal configurations.^27^ The application of these technologies in the field of immune-oncology is discussed in more detail below.

## 3. Microfluidics for solid tumour immunotherapies

Microfluidic systems provide a cost-effective and sample-efficient platform that enables complex *in vitro* models to be formed and easily studied with a variety of microscopy techniques. This is achieved more easily than for *in vivo* models, because they allow manipulation of specific experimental variables for mechanistic studies.^32,27^ Whilst the technology has been used to develop I/O assays for liquid tumours,^6^ its use in studying solid tumours has been less frequent.^7,29^ The approaches used to date have been grouped below according to their application in immune-oncology.

### Cell interaction and migration studies

Several studies have focussed on investigating the interactions between cancer and immune cells (Table 1).

**Table 1:** Summary of microfluidic publications concerning cell interaction/migration studies in I/O. Legend: Ab: antibody, BCG: Bacillus Calmette–Guérin, CAR-T: chimeric antigen receptor T cells, DC: dendritic cell, FPR1: frizzled-related protein, EMT: epithelial–mesenchymal transition, ICB: immune checkpoint blockade, IT: immunotherapy, NK: natural killer cells, NP: nanoparticle, PBMC: peripheral blood mononuclear cells, PDMS: polydimethylsiloxane, TCR: T cell receptor

Author	Topic	IT type	Model	Chip material	2D/3D	Static/perfusion	Chip layout
Hsu *et al.*, 2012 (ref. 33)	Interactions between human lung cancer cells, macrophages and myofibroblasts	General cell interactions/migration	Cell lines	PDMS	2D	Pneumatic conduits and microvalves allowed control over conditioned medium available to each cell type	Three cell culture chambers connected by *y*-shaped channel designed so all angles were at 120° to allow for symmetrical distribution of conditioned media 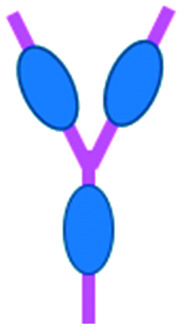
Businaro *et al.*, 2013 (ref. 34)	Role of IRF-8 in communications between cancer and immune cells	General cell interactions/migration	Mixed	PDMS	3D	Static – manual pipetting	Three cell culture chambers connected by an array of microchannels to permit chemical and physical contact amongst the two cell types 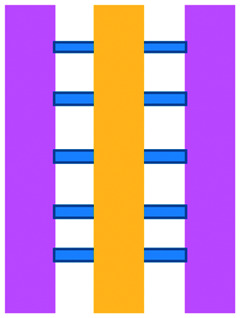
Agliari *et al.*, 2014 (ref. 35)	Benefit of integrating microfluidics with mathematical models to fully quantify experimental image data of real-time interactions between cells	General cell interactions/migration	Mixed	PDMS	3D	Static – manual pipetting	Three cell culture chambers connected by an array of microchannels to permit chemical and physical contact amongst the two cell types 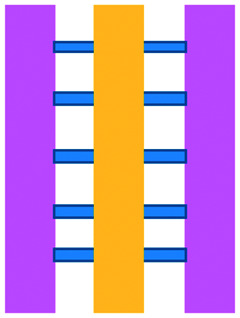
Mattei *et al.*, 2014 (ref. 36)	Role of IRF-8 in communications between cancer and immune cells	General cell interactions/migration	Mixed	PDMS	3D	Static – manual pipetting	Three cell culture chambers connected by an array of microchannels to permit chemical and physical contact amongst the two cell types 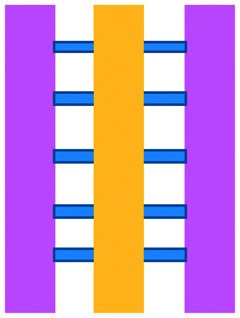
Bai *et al.*, 2015 (ref. 37)	Effect of different macrophage subtypes on tumour aggregate dispersion (mimicking EMT)	General cell interactions/migration	Mixed	PDMS	3D	Static – manual pipetting	Four cell culture chambers connected by an array of microchannels to permit chemical and physical contact amongst multiple cell types and allowing hydrogel formation 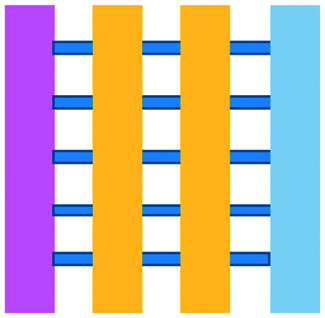
Zhao *et al.*, 2015 (ref. 38)	Role of lactate on macrophage recruitment by and cytotoxicity against cancer cells (relevant to BCG vaccine immunotherapy)	General cell interactions/migration	Cell lines	PDMS	3D	Static – manual pipetting	Four culture chambers with one media channel, each of which could house a different cell type. One matrigel channel and seven migration channels lay between each adjacent culture chamber with each chamber having its own media channel 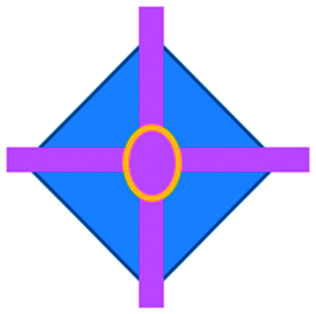
Liu *et al.*, 2015 (ref. 39)	Sensitivity of cancer cells to six different chemotherapy regimes	General cell interactions/migration	Cell lines	PDMS	3D	Microscale vacuum suction apparatus	Culture channels interconnected by microchannels to allow exchange of soluble biological factors and metabolites between cell types. Four cell culture areas connected to a central pool through microchannels which functioned to provide a pressure balance during matrigel perfusion 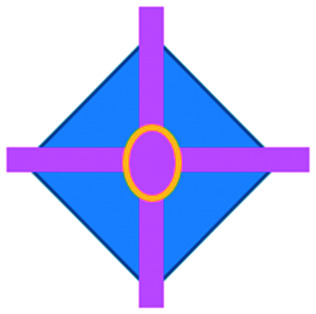
Vacchelli *et al.*, 2015 (ref. 40)	Effect of FPR1 expression on DC response to cancer cells after chemotherapy treatment	General cell interactions/migration	Mixed	PDMS	3D	Static – manual pipetting	Three cell culture chambers connected by an array of microchannels to permit chemical and physical contact amongst the two cell types 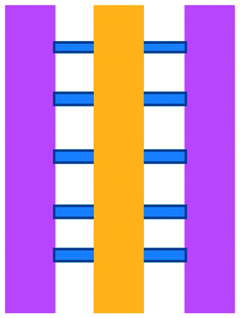
Biselli *et al.*, 2017 (ref. 41)	Interactions between human breast and colon cancer cells and human PBMC	General cell interactions/migration	Mixed	PDMS	3D	Static – manual pipetting	Three cell culture chambers connected by an array of microchannels to permit chemical and physical contact amongst the two cell types 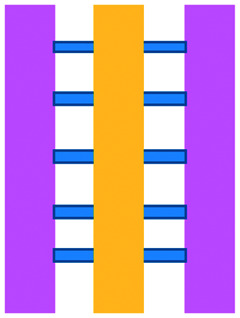
Lucarini *et al.*, 2017 (ref. 42)	Effect of the drug decitabine (DAC) in enhancing anti-tumour effects of IFN through immune cell recruitment to the tumour site	General cell interactions/migration	Mixed	PDMS	3D	Static – manual pipetting	Three cell culture chambers connected by an array of microchannels to permit chemical and physical contact amongst the two cell types 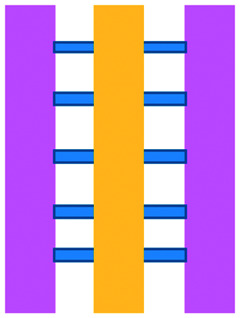
Chen *et al.*, 2018 (ref. 43)	Role of inflamed neutrophils in promoting cancer cell metastasis under perfusion conditions	General cell interactions/migration	Mixed	PDMS	3D	Perfusion of vascular network – manual pipetting	Formation of 8 independent vascular beds with a single gel injection port connected by a branching network. Each sub-unit consisted of 4 parallel channels 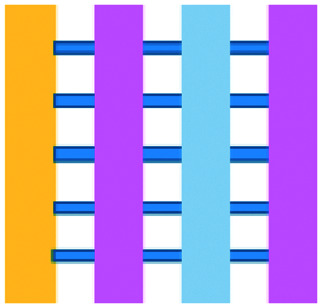
Boussommier-Calleja *et al.*, 2019 (ref. 44)	Migration and development of various subsets of monocytes and monocyte-derived macrophages as targets for anti-metastatic immunotherapies and their effect on cancer cell extravasation	General cell interactions/migration	Cell lines	PDMS	3D	Perfusion of vascular network – manual pipetting	Three parallel channels where monocytes can be observed over a 5 day period migrating through an endothelial barrier to interact with fibroblasts in a central hydrogel channel 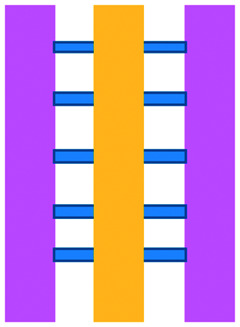
Lei *et al.*, 2020 (ref. 45)	Interactions between cancer and immune cells involved in tumour escape from immune surveillance	General cell interactions/migration	Mixed	Paper layer on top of PMMA layer	3D	Static – manual pipetting	Paper layer containing 5 microreactors on top of a PMMA layer with hydrogel diffusion channels 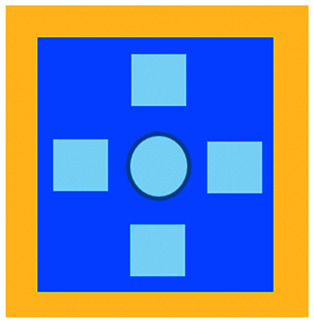

**Fig. 2:**
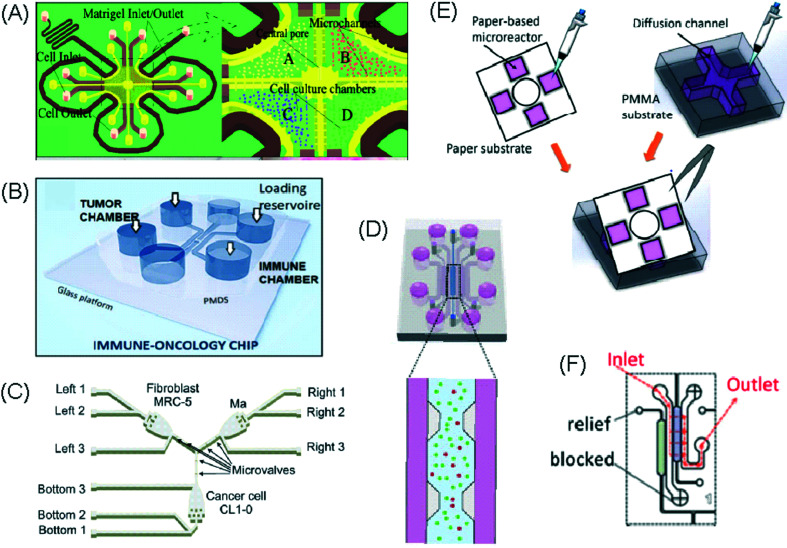
Microfluidic assays for studying cancer and immune cell migration and interactions. (A) Adapted with permission from Liu *et al.*, 2015, Copyright 2015, *Oncotarget*.^39^ (B) Adapted with permission from Biselli *et al.*, *Scientific Reports*, 2017 http://creativecommons.org/licenses/by/4.0/. (C) Adapted with permission from Hsu *et al.*, 2012, Copyright 2012, *Integrative Biology*.^33^ (D) Adapted with permission from Boussommier-Calleja *et al.*, 2018, Copyright 2018, Elsevier.^44^ (E) Adapted with permission from Lei *et al.*, 2020, Copyright 2017, American Chemical Society.^45^ (F) Adapted with permission from Chen *et al.*, 2018, Copyright 2018, National Academy of Sciences.^43^

Mattei *et al.* developed a microfluidic device to study the role of the IRF-8 transcription factor in cross-talk between murine melanoma and immune cells and the release of soluble factors resulting in immune cell infiltration.^36^ The device was composed of an enclosed central microfluidic chamber with externally accessible compartments on either side for culturing adherent B16.F10 murine melanoma cells and non-adherent splenocytes from wild type (WT) and IRF-8 KO mice. Compartments were connected by an array of microchannels that permitted chemical and physical contact amongst the two cell types. It was observed that tumour cells were more invasive when cultured *in vitro* alongside IRF-8 KO splenocytes rather than WT splenocytes. Consistently with *in vivo* findings, this suggests IRF-8 inhibits tumour cell invasion. A previous study by Businaro *et al.* also revealed, through FACS analysis, an upregulation of CD69, a marker for activation, for WT splenocytes.^34^ Further investigating the interaction between WT/IRF-8 KO immune cells and murine B16.F10 melanoma cells, Agliari *et al.* created mathematical models and performed data analysis on the dynamic movement of the splenocytes.^35^ More recently, Biselli *et al.* utilised the same platform to study interactions between doxorubicin treated human breast (MDA-MB-231) and colon cancer cells (patient derived and HCT116 cell line) and human PBMC (from healthy donors) expressing different genetic variants of the FPR1 gene, known to influence the response of phagocytic cells (Fig. 2B).^41^ PBMCs sensed the chemo-attract signals from chemotherapy-treated tumour cells and the device provided the ability to record leukocyte migration towards target tumour cells using time-lapse imaging, revealing that wild type PBMCs moved towards tumour cells, whereas mutant variants did not show target engagement. These results mimicked clinical outcomes where patients who were mutant carriers had a poorer prognosis. Other research looked at the role of the FPR1 gene using a microfluidic device where immune cell migration towards and interaction with chemotherapy treated cancer cells could be recorded.^40^ This model was subsequently modified to study PBMC recruitment towards ECM-embedded (Matrigel) cancer cells exposed to decitabine (DAC) and/or IFN.^42^ Greater infiltration of PBMC was reported toward tumour cells treated with both DAC and IFN in comparison to one agent alone and to untreated cells.

Bai *et al.* developed a multi-channel setup where human umbilical vein endothelial cells (HUVEC) were co-cultured with hydrogel-embedded tumour aggregates and macrophages.^37^ The effect of different macrophage subtypes on tumour aggregate dispersion was studied to mimic epithelial–mesenchymal transition (EMT), inducing cancer cell dissociation *via* CD11b and ICAM-1 interaction. The study showed ICAM-1 involvement in the dissociation and migration of tumour cell aggregates. The use of microfluidics in this application allowed real-time monitoring and precise measurements of cell–cell distances. Subsequently, Chen *et al.* studied the role of neutrophils in promoting cancer cell metastasis under perfusion conditions in order to assess their potential as targets for cancer immunotherapies (Fig. 2F).^43^ The device used allowed the formation of eight independent vascular beds with a single gel injection port connected by a branching network. Tumour cells proximal to neutrophils showed significantly higher extravasation, suggesting that close neutrophil association is a determining factor for tumour cell extravasation. More recently, a microfluidic device was proposed to study the migration and development of various subsets of monocytes and macrophages as targets for anti-metastatic immunotherapies and their effect on cancer cell extravasation (Fig. 2D).^44^

Other work in this field includes the development of microfluidic assays to study interactions between human lung cancer cells, macrophages and myofibroblasts (Fig. 2C).^33^ A Y-shaped channel connected three chambers and was designed so all angles were at 120° to allow for symmetrical distribution of conditioned media. The work showed that TNF can simultaneously promote cancer cell migration while also limiting the migration-promoting abilities of myofibroblasts, but did not study the role of anti-TNF antibodies in the context of cancer immunotherapy.

A microfluidic co-culture chip, consisting of an ECM filled channel (Matrigel) and seven migration channels between culture chambers, was created to study the role of lactate on macrophage recruitment and cytotoxicity against cancer cells, which has relevance to BCG immunotherapy.^38^ This was the first report showing that lactate can, on its own, initiate macrophage recruitment and that quercetin, a lactate inhibitor, can halt this action. Reduced cancer cell migration was observed for co-cultures with M1 macrophages but not M2. These findings indicated that M1 macrophages elicit an anti-metastatic action on tumour cells that is consistent with clinical results reporting that BCG-induced M1 polarization of macrophages inhibited progression and metastasis of transitional cell carcinoma of the bladder (TCCB). Results indicated that lactate was able to reprogram macrophages from pro-tumour cells to anti-tumour cells and to reduce cancer cell viability when in co-culture with macrophages. The quantitative and dynamic observation of macrophage migration and behaviour could not have been achieved with conventional methods, such as tube formatting assays and transwell migration assays. A similar device was also used for co-culturing four different cell types to depict a bladder cancer microenvironment using perfusion equipment to deliver continuous flow (Fig. 2A).^39^ The system was composed of a U-shaped channel with ECM (Matrigel) infused media and cell culture channels interconnected by microchannels to allow the different cell types to exchange soluble biological factors and metabolites. The sensitivity of cancer cells to six different chemotherapy regimens was assessed to study *in vitro* the effects on macrophage migration to the tumour compartment.

The role of nanoparticles (NPs) in immunotherapeutic cell interactions has also been investigated. Chemokine-loaded folic-acid conjugated NPs targeting folic-acid receptor expressing cancer cells were studied in a work by Wimalachandra *et al.*^46^ A device was designed to test the potential of chemokine-loaded NPs to elicit the migration of DC and T cells through an endothelial barrier towards cancer cells. Quantification of DC and T cells showed a greater presence of these cells after administration of chemokine-loaded folic-acid NP in comparison to folic-acid NP only.

Alternative device materials have also been used for immunotherapy studies in microfluidics. A novel microfluidic device was made of a paper layer containing five microreactors on top of a PMMA layer with hydrogel diffusion channels (Fig. 2E).^47^ The device was developed to better understand the interactions between cancer and immune cells involved in tumour escape from immune surveillance. The chip allowed study of crosstalk between different cell types which is difficult using traditional Petri-dishes or well-plates. It can also be used to carry out neutralising and competitive assays. Paper-based microfluidic devices are cost-effective and can maintain oxygen and nutrient gradients that depict organ-level functions.

### Immune cell mediated cytotoxicity studies

The most commonly studied form of immunotherapy using microfluidic technology is ACT therapy (Table 2).

**Table 2:** Summary of microfluidic publications concerning immune cell mediated cytotoxicity studies. Legend: Ab: antibody, ADCC: antibody-dependent cellular cytotoxicity, BCG: Bacillus Calmette–Guérin, CACI-IMPACT: cytotoxicity assay for cancer immunotherapy, CAR-T: chimeric antigen receptor T cells, CTL: cytotoxic T lymphocytes, DC: dendritic cell, FPR1: frizzled-related protein, EMT: epithelial–mesenchymal transition, HBV: hepatitis B virus, HUVEC: human umbilical vein endothelial cells, ICB: immune checkpoint blockade, IDES: interdigitated electrode structures, ITO: indium tin oxide, IT: immunotherapy, OET: optoelectronic tweezers, OPD: organic photodiode, NK: natural killer cells, NP: nanoparticle, PBMC: peripheral blood mononuclear cells, PC: polycarbonate, PEG-DA: poly(ethylene glycol) diacrylate, PDMS: polydimethylsiloxane, TCR: T cell receptor, TiOPC: titanium oxide phthalocyanine, ZA: zoledronic acid

Author	Topic	IT type	Model	Chip material	2D/3D	Static/perfusion	Chip layout
Charwat *et al.*, 2013 (ref. 48)	Simultaneous study of tumour cell invasion and escape of immune surveillance. Effect of a nonlethal cytotoxic agent (urine) on adherent cells in relation to the use of urine analysis for non-invasive biomarker detection in diagnoses	T cells	Mixed	PDMS	3D	Perfusion – syringe pump, impedance sensors, notch filter, light scattering sensors, external valves, injection ports	IDES and integrated fully spray-coated organic photodiode OPD arrays for electrical and optical light scattering measurements under perfusion conditions. PDMS layer sandwiched between electronics and upper interface 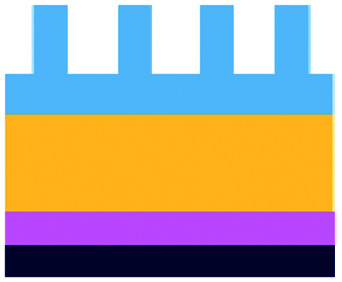
Layer *et al.*, 2017 (ref. 49)	T cell chemotaxis studied *via* tracking migration and speed of T cells through a chemokine gradient	T cells	Mixed	μ-Slide III 3in1 Ibidi plastic	3D	Perfusion – syringe pump	Three cell culture chambers connected by an array of microchannels to permit chemical and physical contact amongst the two cell types 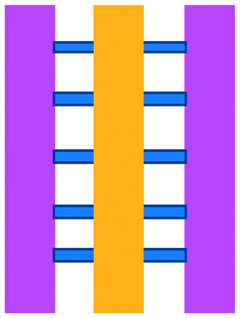
Pavesi *et al.*, 2017 (ref. 51)	Evaluation of T cell function against single tumour cells and aggregates	TCR-engineered T cells	Mixed	PDMS	3D – compared 3D microfluidic with 2D results. 2D assays significantly overestimated T cell killing abilities and could not determine an effect of hypoxia on T cell killing	Static – manual pipetting	Three cell culture chambers connected by an array of microchannels to permit chemical and physical contact amongst the two cell types 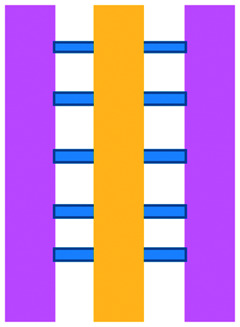
Ayuso *et al.*, 2018 (ref. 53)	NK cell cytotoxicity and ADCC	NK and Abs (anti-EpCam)	Cell lines	PDMS	3D	Static – manual pipetting	Multi-compartments containing spheroids in ECM with an endothelial lined channel on either side to represent vasculature. NK cells either embedded in the gel or perfused through the endothelial channels 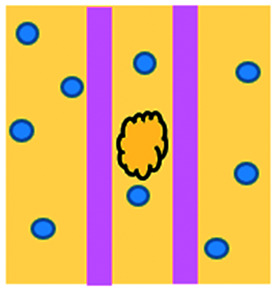
Ke *et al.*, 2017 (ref. 54)	NK cell activity and their interaction with cancer cells	NK	Cell lines	TiOPC coated ITO glass with PEG-DA microwells	2D – TiOPc-based OET	Perfusion – syringe pump	OET cell manipulation into PEG-DA hydrogel within four-leaf-clover-shaped microwells. The electric-field distribution in the device is controlled by a dynamic light pattern, which created the OET non-contact force to guide cell movement 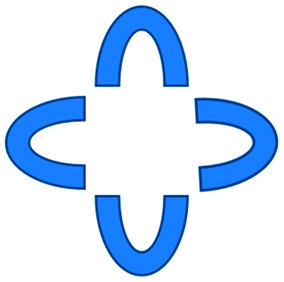
Lee *et al.*, 2018 (ref. 55)	To determine monocytes inhibition of the function of HBV TCR T cells and their dependence on the method of cell engineering to produce the T cells	TCR-engineered T cells	Mixed	PDMS	3D – in comparison, the 2D equivalent culture showed no effect of the monocytes on either cell type	Static – manual pipetting	Three cell culture chambers connected by an array of microchannels to permit chemical and physical contact amongst the two cell types 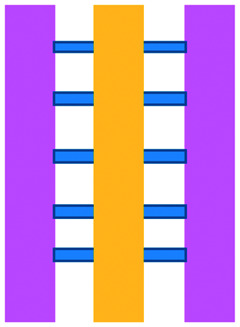
Ando *et al.*, 2019 (ref. 52)	Effect of oxygen availability on the cytotoxicity of CAR T cells	CAR T	Cell lines	PDMS	3D	Static – manual pipetting	PDMS plasma-bonded to a glass slide with a milled PC cap 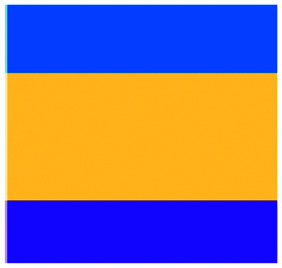
Di Mascolo *et al.*, 2019 (ref. 50)	T cells exposed to ZA containing nanoparticles (ZA-SPNs) to determine their promotion of T cell extravasation and migration towards cancer cells through a vascular structure	T cells	Mixed	PDMS	3D	Perfusion – syringe pump	Two cell culture chambers connected by an array of microchannels to permit chemical and physical contact amongst the two cell types 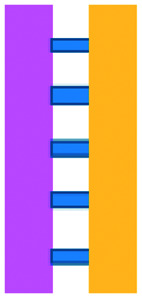
Park *et al.*, 2019 (ref. 56)	Cytotoxic capabilities of lymphocytes	NK	Cell lines	Polysterene, mass produced from injection moulding	3D–3D experiments showed significantly less NK cell cytotoxicity compared to 2D	Perfusion of vascular network – manual pipetting	Rail-based microstructures with hydrophilic surfaces for gel patterning 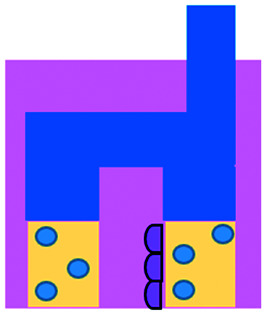
Wu *et al.*, 2019 (ref. 57)	Droplets solidified in CaCl_2_ solution to form porous microspheres that could be used as a vehicle to house NK-92MI cells for immunotherapeutic applications	NK	Cell lines	Microfluidic electrospray forming PEO/ALG droplets	3D – precursor solution of alginate solution and PEO injected through an electrospray microfluidic device under an electric field to form droplets	Microfluidic electrospray	Microfluidic electrospray 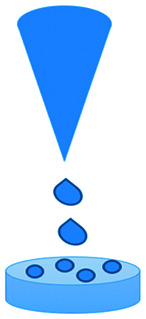
Chen *et al.*, 2020 (ref. 58)	Migration characteristics and anti-cancer response of CTLs	T cells	Cell lines	PDMS	3D	Perfusion – syringe pump	Three cell culture chambers connected by an array of microchannels to permit chemical and physical contact amongst the two cell types 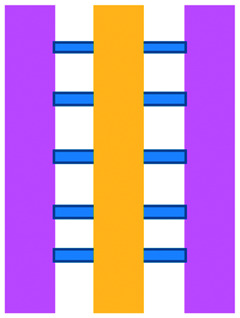
Ayuso *et al.* 2021 (ref. 59)	NK cell cytotoxicity and ADCC	NK, small molecule inhibitors and antiPD-L1 Ab	Mixed	PDMS	3D	Static – manual pipetting	Collagen hydrogel containing tumour cells injected. Endothelial lined channel representing vasculature for NK cells, antibodies and inhibitors to be perfused through 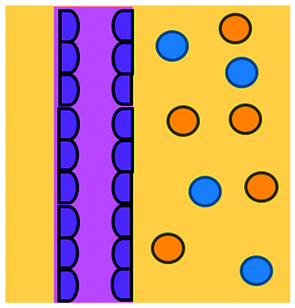

**Fig. 3:**
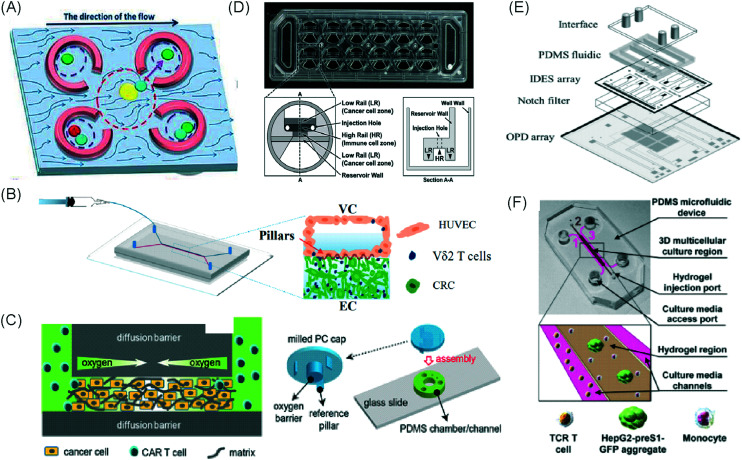
Microfluidic examples of immune cell mediated cytotoxicity. (A) Adapted with permission from Ke *et al.*, 2017, Copyright 2017, *Lab on a Chip*.^54^ (B) Adapted with permission from Di Mascolo *et al.*, 2019, Copyright 2019, *Cancers*, http://creativecommons.org/licenses/by/4.0/;^50^ (C) adapted with permission from Ando *et al.*, 2019, Copyright 2019, *Adv. Healthcare Mater.*^52^ (D) Adapted with permission from Park *et al.*, 2019, Copyright 2019, *Frontiers in Immunology*, http://creativecommons.org/licenses/by/4.0/.^56^ (E) Adapted with permission from Charwat *et al.*, 2013, Copyright 2013, *American Chemical Society*.^48^ (F) Adapted with permission from Lee *et al.*, 2018, Copyright 2018, *Frontiers in Immunology*, http://creativecommons.org/licenses/by/4.0/.^55^

An approach based on impedance sensing and optical light scattering was used to monitor interaction amongst tumour, adherent stromal cells and non-adherent immune cells (Fig. 3E).^48^ The platform consisted of interdigitated electrode structures and integrated fully spray-coated organic photodiode arrays for electrical and optical light scattering measurements under perfusion conditions. Cellular impedance sensing was used to detect cell surface interactions while light scattering was used to determine the quantity and morphology of cells present, enabling simultaneous study of tumour cell invasion and escape of immune surveillance. A few years later, a microfluidic device was developed for tracking the migration and speed of T cells through a controlled chemokine gradient,^49^ showing that inhibiting the MYCN (N-Myc) proto-oncogene could improve T cell infiltration. Other work looked at the use of aminobisphosphonates (N-BPs), such as zoledronic acid (ZA), as nanoparticle agents to specifically promote anti-tumour Vδ2 T cell proliferation (Fig. 3B).^50^ The platform enabled study of the dynamics of T cell migration through a vascular bed and across an endothelial monolayer in order to reach tumour cells embedded in hydrogels.

Models looking at the influence of hypoxia and cytokines on ACT therapy have also been reported. A microfluidic device was developed and employed for preclinical assessment of the toxicity of engineered T cells towards tumour cell aggregates embedded in a 3D matrix.^51^ The device was designed for the evaluation of T cell function against single tumour cells and aggregates depending on addition of interferon gamma and TNFα. In this work, outcomes from 3D microfluidic and standard monolayer assays were compared. The 2D assays significantly overestimated T cell killing abilities and were unable to determine an effect of hypoxia on T cell killing. Whereas the 3D cultures found reduced killing of cancer cells at 2% O_2_ compared to 20% O_2_. Increased killing of cancer cells was observed in the presence of inflammatory cytokines, particularly with higher O_2_ conditions. In a different work, Ando *et al.* established a microfluidic protocol integrating an oxygen gradient to study CAR-T cell behavior under a hypoxic gradient compared to normoxic conditions (Fig. 3C).^52^ Results showed that the highest cytotoxicity occurred in normoxic conditions with PD-L1 surface expression of T cells increasing in hypoxic conditions.

In order to determine if monocytes inhibit the function of engineered T cells (as they do with natural T cells, through PD-L1/PD-1 signalling), human primary monocytes were used to mimic the hepatitis B virus (HBV)-related hepatocellular carcinoma tumour microenvironment (Fig. 3F).^55^ T cell cytotoxicity to HepG2 cancer spheroids was found to be impaired by monocytes for retrovirally transduced T cells only and not for mRNA electroporated T cells. No such effect on either T cell type was observed in equivalent 2D assays.

NK cells were also recently investigated with microfluidic studies. Ayuso *et al.* designed a multi-chamber microfluidic device to assess NK cell cytotoxicity and antibody-dependent cellular cytotoxicity (ADCC).^53^ NK cells were either embedded in hydrogel alongside cancer spheroids or perfused through endothelial channels, so that NK and antibody penetration in 3D and through the vasculature could be observed. Z-Stack images showed antibodies administered into side channels were able to extravasate from the endothelial layer and penetrate the ECM, but not the spheroids. NK cells, however, were able to home in on tumour spheroids located hundreds of microns away and to successfully infiltrate and kill tumour cells on the periphery of a spheroid, as well as in its core. More recently, the same group adapted the microfluidic set-up to investigate NK exhaustion due to tumour environmental stresses reproduced on-a-chip.^59^ The device design allowed to control nutrient and pH gradients across 3D tumour models, as well as inducing cell proliferation and necrosis. Gene expression analysis showed greater exhaustion of the NK cells cultured in devices in comparison to those in well plates. However, this effect could be partially negated with the use of an anti-PD-L1 antibody, atezolizumab, and IDO-1 inhibitor, epacadostat. Results demonstrated suppression of NK cell cytotoxicity for regions where MCF7 cells were able to create environmental gradients supportive of their proliferation. In contrast, patient derived breast cancer cells were unable to elicit sufficient environmental stress to be detrimental to NK cell cytotoxicity.

Further, the use of titanium oxide phthalocyanine (TiOPc)-based optoelectronic tweezers was established for controlling cell–cell contacts to study immune and cancer cell interactions (Fig. 3A).^54^ Single cells were manipulated into PEG-DA hydrogel four-leaf-clover-shaped microwells, where direct cell-to-cell contact could occur. The benefit of this model was that it avoided continuous fluid flow and shear stress in the specific device regions where secreted proteins would be washed out and interactions between cells and NK cell activity were impacted. Park *et al.* proposed a 3D cytotoxicity assay to determine cytotoxic capabilities of lymphocytes (Fig. 3D).^56^ Polystyrene devices were mass produced using injection moulding to form plastic culture arrays and rail-based microstructures with hydrophilic surfaces. The multi-well format allowed multiple assays to be performed in one device simultaneously. Another example of microfluidic immunotherapeutic application to study NK behaviour was provided by Wu *et al.* who reported a setup to produce alginate microspheres to protect NK cells from the TME and from being rejected by the host's immune system when injected.^57^ Microspheres were shown to keep more than 85% of cells viable after 72 h with cells remaining viable for up to 14 days. Cytotoxic factors, perforin, granzyme and IFN-γ were shown to be released from the cells over a 7 day period. NK cells encapsulated in microspheres showed greater killing *in vivo* in comparison to free NK cells with the microsphere injections not resulting in any adverse side effects *in vivo*.

### Mechanistic and mode of action studies

Other immunotherapy-related approaches have also been investigated in microfluidics (Table 3).

**Table 3:** Summary of microfluidic publications concerning mechanistic and mode of action studies I/O. Legend: Ab: Antibody, BCG: Bacillus Calmette–Guérin, CAF: cancer associated fibroblasts, CAR-T: chimeric antigen receptor T cells, COP: cyclo olefin polymer, CTC: circulating tumour cells, DC: dendritic cell, FPR1: frizzled-related protein, EMT: epithelial–mesenchymal transition, ICB: immune checkpoint blockade, IFNα: interferon alpha, IT: immunotherapy, MDOTS: murine-derived organotypic tumour spheroids, NK: natural killer cells, NP: nanoparticle, PBMC: peripheral blood mononuclear cells, PDMS: polydimethylsiloxane, SCC: squamous cell carcinoma, TCR: T cell receptor, TIL: tumour-infiltrating lymphocytes, TME: tumour microenvironment

Author and ref no.	Topic	IT type	Model	Chip material	2D/3D	Static/perfusion	Chip layout
Zervantonakis *et al.*, 2012 (ref. 64)	Examining the mode of action by which macrophages influence tumour cells *via* TNF release	Ab	Mixed	PDMS	3D	Static – manual pipetting	Three cell culture chambers connected by an array of microchannels to permit chemical and physical contact amongst the two cell types 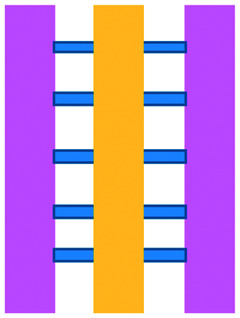
Lu *et al.*, 2015 (ref. 65)	DC/tumour fusions to elicit anti-tumour immunity	DC vaccine	Cell lines	PDMS	3D	Perfusion – syringe pump, electrodes	960 pairs of trapping channels. Cell electrofusion device that can pair and fuse homogeneous and heterogeneous cells 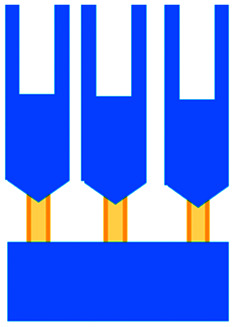
Jenkins *et al.*, 2017 (ref. 66)	Novel TBK1/IKKε inhibitor mechanisms	ICB	Primary	PDMS	3D	Static – manual pipetting	Three cell culture chambers connected by an array of microchannels to permit chemical and physical contact amongst the two cell types 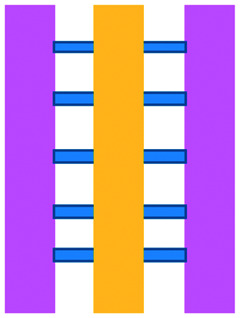
Kulasinghe *et al.*, 2017 (ref. 67)	Non-invasive method to identify candidates for anti-PD-L1 therapy. Involved blood sample from a SCC patient to determine the PD-L1 expression of CTCs	ICB	Mixed	PDMS	3D	Perfusion – syringe pump	Spiral microfluidic channel 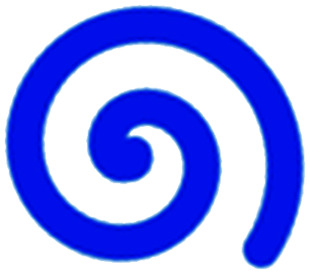
Parlato *et al.*, 2017 (ref. 68)	Effect of biochemical stimuli on DC migration. IFNα-conditioned dendritic cells for use as a therapeutic vaccine in combinations with romidepsin	DC vaccine	Cell lines	PDMS	3D	Static – manual pipetting	Five cell culture chambers connected by an array of microchannels to permit chemical and physical contact amongst the two cell types 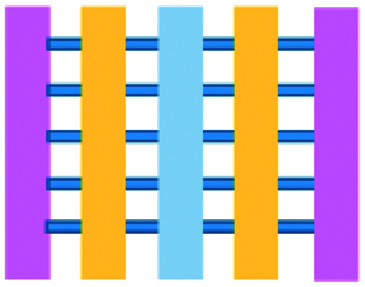
Aref *et al.*, 2018 (ref. 62)	ICB in conjunction with small hydrophobic molecules	ICB	Primary	COP plastic device from AIM BIOTECH	3D – MDOTS viability affected by PD-1 blockade in 3D microfluidic culture but not in 2D culture using 384-well plates	Static – manual pipetting	Three cell culture chambers connected by an array of microchannels to permit chemical and physical contact amongst the two cell types 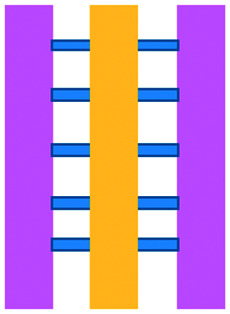
Huh *et al.*, 2018 (ref. 69)	Mimicking the drug toxicity-induced pulmonary oedema seen in cancer patients after IL-2 treatment	Cytokines-IL-2	Cell lines	PDMS	2D	Perfusion – syringe pump, vacuum pump	Two parallel microchannels separated by a thin and porous ECM coated membrane, permitting perfusion and cyclic stretching of the cell layers attached to a flexible membrane, mimicking physiological breathing motions 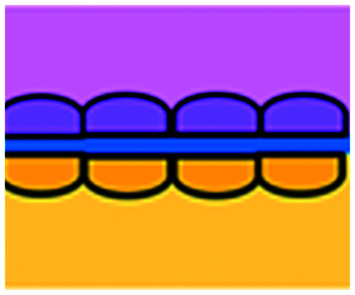
Moore *et al.*, 2018 (ref. 63)	Study of the mechanisms by which anti-PD-1 antibodies augment the cytotoxicity of TILs	ICB	Primary	COC plastic EVIDENT device	3D	Perfusion – pressure-pump driven system	Tumour fragment trapped in V-designed channels 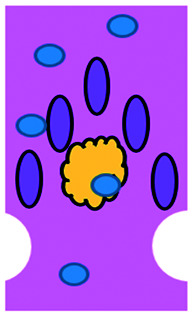
Nguyen *et al.*, 2018 (ref. 70)	Effects of trastuzumab and CAF on cancer cell proliferation, cell death and motility. Effects of co-culture with CAF and immune cells in 3D	Ab	Mixed	PDMS	3D – the drug decreased mitosis, tumour growth and apoptosis. In 2D experiments the drug did not inhibit the growth of cancer cells	Perfusion – syringe pump	Five cell culture chambers connected by an array of microchannels to permit chemical and physical contact amongst the two cell types 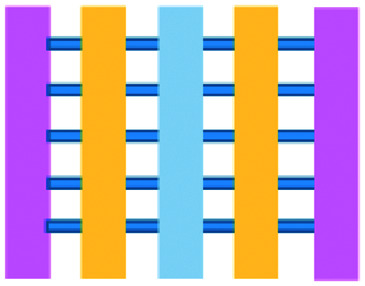
Yin *et al.*, 2018 (ref. 71)	Antibodies for the identification of the stage of cancer progression and determination of the optimum course of treatment	Ab	Mixed	PDMS and patterned nickel micropillar substrate	3D	Syringe pump and magnets used to immobilize antibodies onto micropillars of device	Chaotic mixer with a patterned nickel micropillar substrate 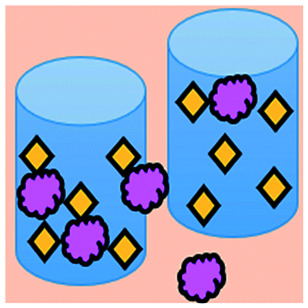
Wimalachandra *et al.*, 2019 (ref. 46)	Chemokine-loaded folic-acid conjugated NPs for targeting folic-acid receptor expressing cancer cells and attracting immune cells towards the target cells	Chemokine-loaded NPs	Mixed	PDMS	3D	Static – manual pipetting	Three cell culture chambers connected by an array of microchannels to permit chemical and physical contact amongst the two cell types 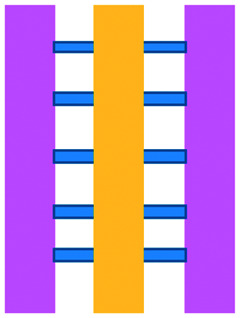

A microfluidic platform has been developed and used for examining the role of macrophages in tumour cell intravasation.^60^ Greater tumour cell intravasation and endothelial permeability was reported when tumour cells were co-cultured with macrophages. This could be reduced by administering anti-TNF antibodies. A device originally used to study angiogenic growth was employed by Jenkins *et al.* to test a novel TBK1/IKKε inhibitor on murine-derived organotypic tumour spheroids (MDOTS) and patient-derived organotypic tumour spheroids (PDOTS).^61^ Greater cell death of MC38 MDOTS was seen when the inhibitor was combined with anti-PD-1 treatment and was dose- and time-dependent.

The use of plastic microfluidic devices made using cyclic olefin polymer (COP) have also been proposed by Aref *et al.* for testing of immune checkpoint blockade (ICB) in conjunction with small hydrophobic molecules, as these can be adsorbed by PDMS.^62^ Murine- and patient-derived organotypic tumor spheroids from a patient with a small intestinal neuroendocrine tumour (SI-NET) were treated with ICB treatment with anti-PD-1 + anti-CTLA-4 in combination and as monotherapy. Dual ICB showed greater immune-mediated killing and relative expansion of CD8 T cells and macrophages in comparison to control and single agent ICB-treated PDOTS. Biopsy fragments were also studied in microfluidics developing an *ex vivo* immuno-oncology dynamic environment (Fig. 5F).^63^ This proof-of-concept system allowed culture of up to 12 individual tumour biopsy fragments subjected to flowing tumour-infiltrating lymphocytes (TIL) in a pressure-pump-driven system. This set-up was utilised in ICB studies and reported increased tumour killing seen in channels with TILs treated with anti-PD-1 ICI. However, issues with fluid obstruction due to bubble formation and cell debris accumulation were recorded.

**Fig. 4:**
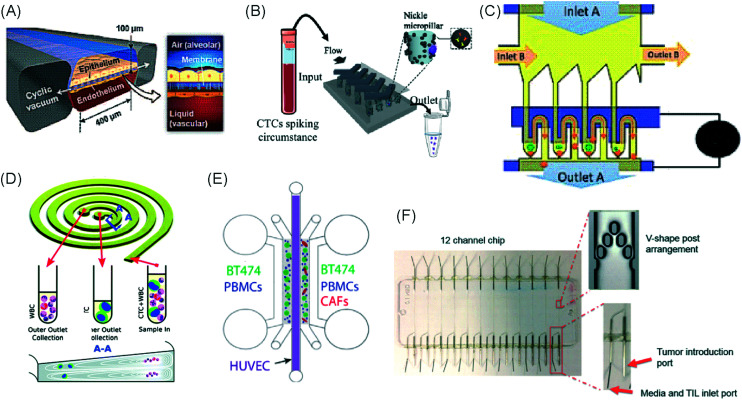
Example of microfluidic technologies for mechanistic and mode of action studies in I/O. (A) Adapted with permission from Huh *et al.*, 2018, Copyright 2018, *Science Translational Medicine*.^69^ (B) Adapted with permission from Yin *et al.*, 2018, Copyright 2018, *American Chemical Society*.^71^ (C) Adapted with permission from Lu *et al.*, 2015, Copyright 2015, *Oncotarget*, http://creativecommons.org/licenses/by/4.0/.^65^ (D) Adapted with permission from Warkiani *et al.*, 2014, Copyright 2014, *The Royal Society of Chemistry*, http://creativecommons.org/licenses/by/4.0/.^72^ (E) Adapted with permission from Nguyen *et al.*, 2018, Copyright 2018, *The Royal Society of Chemistry*, http://creativecommons.org/licenses/by/4.0/.^70^ (F) Adapted with permission from Moore *et al.*, 2018, Copyright 2018, The Royal Society of Chemistry.^63^

DC studies have additionally been carried out using microfluidics. Parlato *et al.* investigated IFNα-conditioned dendritic cells for use as a therapeutic vaccine in combinations with chemotherapy drug, romidepsin.^68^ Migration channels connected the tumour and immune chambers to allow analysis of DC migration, phagocytosis and cell–cell interactions, aiming to mimic *in vivo* DC crossing of the endothelial barrier. A cell electrofusion device that can pair and fuse homogeneous and heterogeneous cells was produced by Lu *et al.* and is relevant to the production of DC-tumour fusion vaccines to elicit anti-tumour immunity (Fig. 4C).^65^ The device consisted of 960 pairs of trapping channels with 68% and 64% pairing and fusion efficiencies, respectively. Fused cells could be easily extracted from the device, in comparison to other electrofusion systems. The use of microfluidics in cell fusion offers several other advantages over conventional methods including: precise pairing of cells, greater fusion efficiencies and cell viability and reduced sample contamination and Joule heating effects.

Microfluidic work investigating immunotherapeutic antibodies has also been reported. Microfluidic assays were developed controlling cell composition in experiments carried out with and without the drug, trastuzumab, a monoclonal antibody against the HER2 receptor (Fig. 4E).^70^ The device enabled direct visualization and quantification of proliferation, cell death and motility, including the influence of co-culture with CAF and immune cells in a 3D environment. The drug was shown to decrease mitosis, tumour growth and apoptosis with the extent of these anti-tumour effects varying depending on the composition of cells present in the culture. In contrast, 2D experiments showed that the drug did not inhibit the growth of these cancer cells.

Alternative applications for immunotherapy studies involving microfluidics have included a setup that functioned to mimic the drug toxicity-induced pulmonary oedema that is seen in cancer patients after IL-2 treatment (Fig. 4A).^69^ The platform hosted alveolar epithelial cells exposed to air flow and interfaced with an IL-2 perfused endothelial cell compartment. Cyclic stretching of a flexible membrane mimicked physiological breathing motions. This device offers the advantage of a greater level of physiological relevance than standard static models. Phase contrast microscopy showed fluid leakage into the alveolar compartment that gradually reduced the air volume.

Kulasinghe *et al.* reported a method to identify potential candidates for PD-L1 therapy.^67^ This involved a microfluidic chip with a spiral channel utilizing Dean flows^72^ (Fig. 4D) to isolate circulating tumour cells (CTC) from blood of patients with squamous cell carcinoma. Extracted CTCs where characterised for PD-L1 expression. Yin *et al.* established a dual-antibody-functionalised microfluidic chaotic mixer using anti-EpCAM and anti-PSMA antibodies immobilized onto micropillars (Fig. 4B)^71^ where captured CTCs could be analysed and subsequently recovered from the device. These approaches enable valuable tools to enrich CTC samples and especially offers the potential for personalized therapy by allowing the identification of the stage of cancer progression and determination of the optimum course of treatment for individual patients.

## 4. Discussion

**Fig. 5:**
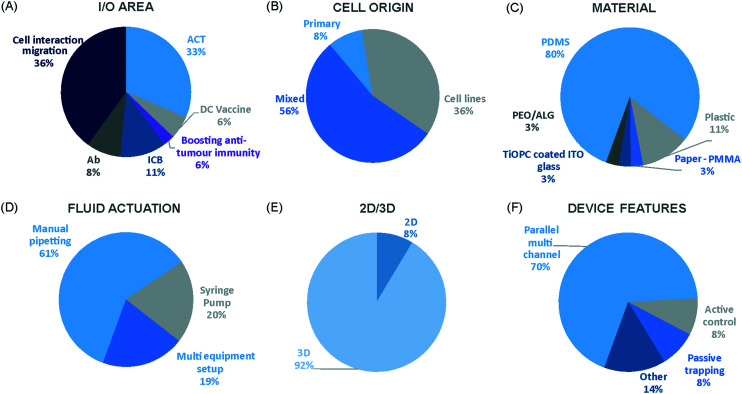
Characterization of microfluidic approaches for *in vitro* studies of immunotherapy for solid tumours. Pie charts showing: (A) I/O area or type of immunotherapy implemented, (B) the origin of the cell used for *in vitro* assays, (C) the bulk material of the microfluidic device, (D) the type of fluid actuation, (E) the spatial arrangement of the cell model and (F) the features of the device that characterised or enabled the microfluidic model.

We have charted the studies reported above according to aspects that we consider being of particular importance for broadening the use of microfluidics in immunotherapy studies: the broad area of immune-oncology investigated or type of immunotherapy being implemented, the microfluidic cell model and its complexity, the constituent material of the microfluidic chip, the type of fluid actuation used to interface with the microfluidic device and the device design (Fig. 5).

The majority of these studies focused on investigating cell-to-cell interactions and cell migration (36%) in a simplified reconstructed TME, rather than looking at the effects of specific immunotherapy agents. Of the actual immunotherapy regimes tested, ACT therapies (33%) were the most studied, followed by ICB (11%), antibodies (8%), DC vaccines (6%) and cytokines/chemokines (6%). Immune cell mediated cytotoxicity has increasingly generated interest in recent years and has seen success in a range of haematological malignancies, potentially driving the development of microfluidic assays in this area. Overall, the miniaturised environment and the enhanced control over cell positioning obtained in microfluidics is inherently suitable for building advanced complex models that are not easily achievable with off-chip techniques. Therefore, it is not surprising that over two thirds of the models implemented ACT studies and cell-interaction/migration investigation, these being considerably more challenging to image *in vivo* and to recreate in standard well plate systems. One major advantage of microfluidic models is that these allow for a reductionist approach by focusing on one area of study and eliminating other variables. Multiple cell types can be characterized and their behaviour changes investigated depending on which cytokines or other cell types are present in an assay. An open question remains around why other immunotherapies have not been investigated in microfluidic formats to date, possibly due to the complexity of the model required to simulate systemic response when testing vaccines or non-specific immune-stimulation.

Over a third of the papers considered in this review relied solely on immortalized cell lines. Over half of the studies used a combination of primary cells and cell lines, while only 8% used primary tissue alone. Although using primary tissue increases the complexity of the assays (due to heterogeneity and potential change in cell phenotype) and the cost, it does enhance their translational value. As a number of these studies were proof-of-concept work, it could be argued that using primary cells would have been an unnecessary complication, as cell lines guarantee an easier approach to illustrate the operation of the device and validate the assay initially. Nonetheless, it is essential that follow-up studies emerge showing outcomes that go beyond the scope of proving feasibility of the microfluidic technology alone.

Generally speaking, microfluidic devices are fabricated using various polymeric materials, from elastomers to thermoplastics, but also paper and glass as substrates.^73^ Within the cohort of papers reviewed, the vast majority (80%) of the devices were fabricated in polydimethylsiloxane (PDMS), with few reports of thermoplastic, paper and glass microsystems being used. Soft-lithography is abundantly used in research laboratories, this involves the fabrication of microstructures *via* replica moulding from master templates, most of which are created by photolithography.^74^ The most popular material option for this is PDMS, due to its favourable bio-chemical properties and lower cost compared to alternative materials.^75^ Its optical transparency allows straightforward visualisation, while its gas permeability facilitates cell culture.^75,76^ It can be adjusted to obtain the desired degree of elasticity, using cross-linking agents.^77^ The flexibility of PDMS allows for its use in a wide range of applications. However, PDMS also presents disadvantages such as bulk absorption of compounds, especially important for compound testing,^73^ and mass production of PDMS based devices is less straightforward than thermoplastic-based manufacturing. Glass and silicon microfluidic devices are more expensive and time consuming to process.^78^ However, glass is a durable material and presents less risk of drug absorption than PDMS.^73^ Thermoplastics, such as polycarbonate and polystyrene, can be readily oxidised to decrease their hydrophobicity for use in microfluidics,^79^ and are also better characterized for drug absorption compared to PDMS, but microfabrication of small features and bonding of several layers are not straightforward processes compared to soft-lithography.^73^ Overall, the material chosen for the device has considerable implications for the intended application of the microfluidic assay. Importantly, interfacing of microfluidic structures, harbouring 3D cellular models, with automated imaging techniques and novel image analysis algorithms can play a fundamental role in large throughput applications and in the standardization of device features.

The majority of the works under consideration used fluid actuation equipment, with 61% of the microfluidic devices relying on manual pipetting alone, whilst 20% were connected to syringe pumps to achieve flow perfusion. A further 19% of papers required a multi-equipment system. Syringe, pneumatic, or peristaltic pumps are commonly used to provide a controlled, continuous delivery of nutrients to cells *via* injection or perfusion using tubing connected to microfluidic devices. The use of cumbersome, tubing-based external instrumentation often poses challenges to the development of the technology for high throughput applications.^44^ Requirement for automated perfused cell culture is typically the main driver in deciding whether continuous connection to external equipment is required. Other solutions are available that can be implemented with open well-based microfluidics, such as interfacing with robotic dispensers,^80,81^ plate rockers^82^ or bespoke microfluidic layouts that can achieve long-lasting, self-generated perfusion.^83,84^

Finally, yet importantly, a predominant structure emerged regarding the layout of the microfluidic device used, supporting cell interaction and migration studies. Individually addressable, parallel multi-chamber designs (used in ∼70% of the studies, Fig. 5E) allowed initial compartmentalization of cellular subtypes in adjacent microenvironments, followed by the development of chemical and cellular communication across chambers *via* microchannel networks or hydrogel barriers. This is possibly the biggest advantage that the microfluidic mesoscale can offer with respect to other techniques. It is able to create complex *in vitro* multicellular models in 3D in a controlled manner, enabling steep molecular gradients to be formed, cell chemotactic mechanisms to be reproduced and different viscous fluids (*i.e.* media and hydrogels) to be used concurrently, recreating several TME characteristics and offering the possibility to study the interactions between various immune, stromal and tumour cell types. The miniaturised scale of microfluidic devices can also provide opportunities to perform such assays using limited patient derived samples. However, areas for improvement remain and the technology requires further development for use in assays involving a broader range of immunotherapies.

Importantly, many of these studies have also characterised the discrepancy between performing the same assay in 2D *vs.* 3D,^51,55,56,62^ further highlighting how more advanced *in vitro* models can be created within the microfluidic environment. *In vitro* models are often considered too simplistic in their depiction of the TME.^85^ This is certainly the case where 2D, cost-effective cell monolayers are used.^30,86^*In vitro* 3D models using *ex vivo* human tumour tissues,^87^ such as spheroids and organoids,^88^ are increasingly being used, but the immune-component, vasculature and other stromal components of such models are often neglected.^89,90^

The lack of *in vitro* models that adequately represent the native *in vivo* environment remains a significant hurdle in the preclinical development of immunotherapy.^91^*In vivo* animal models, can offer greater complexity and a greater degree of physiological relevance than standard *in vitro* models. Various types of murine models are commonly employed, including immunodeficient (*i.e.* lack of an intact immune system) and immunocompetent (*i.e.* presenting an intact immune system) mice. Human cancer cell lines and primary cancer patient tumour tissue can be engrafted into immunodeficient mice, but these lack adaptive immune cells and therefore may fail to fully recapitulate immune responses observed in immunocompetent cancer patients.^3^ Many studies have also reported issues with successful engrafting of human cells and their long-term survival in the host. While cytokines can be administered in an attempt to prolong human cell survival in these models, this has the potential to skew the immune response and could lead to the acquisition of data that is not representative of the actual human immune response.^92,93^ Animal studies are thought to overestimate the benefit of investigated treatments by approximately 30%, with less than 8% of *in vivo* assays able to be successfully translated into clinical cancer trials.^93^ These methods also have the disadvantage of the high costs of experiments, in addition to ethical concerns.^94^ Advanced *in vitro* I/O models could avoid many of the drawbacks of animal experimentation.

The adoption of microfluidic models into the pharmaceutical industry could play a significant role during drug development. Chip-based, advanced and complex *in vitro* models can potentially reduce and, in some cases, replace less predictive 2D assays and more expensive animal models, providing a scalable and versatile platform for immunotherapy development.^95,96^

## 5. Conclusion and future opportunities

**Fig. 6:**
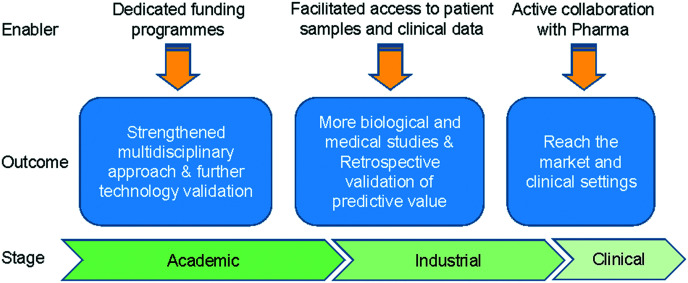
Roadmap to success. The path to validate microfluidic technologies for immunotherapy development.

Overall, microfluidic technologies (including lab-on-a-chip, organ-on-a-chip and microphysiological systems) have the potential to offer solutions that can one day outperform current *in vitro* and animal models, improving our understanding of I/O mechanisms and creating predictive and efficient tools for the cost-effective development of immunotherapies. Looking at the next 10 years, considerable challenges have to be overcome to achieve these objectives (Fig. 6).

Most of the studies described in section 3 focussed on investigating mechanisms of action and represented proof-of-principle studies related to immunotherapies. Therefore, further evidence is needed to demonstrate the full potential of microfluidic assays for testing efficacy of immunotherapies. Follow-up studies are required that use the technology to drive the medical or biological investigation, which will in turn optimise the technology and define specific applications. Technology transfer and increased multidisciplinary collaborations are key aspects to achieve such aims. Alongside this is the need for funding programmes that can sustain diverse and large teams working collectively towards the common goal. Increasing model complexity and data throughput will require side-by-side advances in the automation of the microfluidic protocols, as well as in the development and application of machine learning algorithms and high-level data interpretation. As well as a broad academic team, there is a need for collaboration with Pharma industry and the clinical sector.

It is expected that successful validation of organ-on-a-chip and microfluidic technologies for a specific immune cell type will drive its use to study anti-cancer effects on the tumour in the context of various other immune cells. This will facilitate mechanistic studies at a lower cost than *in vivo* animal experiments, with considerable advantages for straightforward interfacing to high content imaging equipment. Building upon the current microfluidic assays developed for ACT therapy and cell interaction/migration highlighted in this review, we envisage that the technology can be used to develop new assays to identify and isolate immune cells from patient derived tissue and test the efficacy and specificity of T cells (including CAR-T cells). Additionally, the technology could be used to assess the efficacy and penetration of oncolytic virus and antibodies into 3D *in vitro* human models. Finally, the development of body-on-a-chip platforms, where simplified systemic aspects of the immune system could be modelled, represent challenging but rewarding new areas that would benefit those immunotherapies currently under-investigated in microfluidics experiments.

Miniaturisation makes it feasible for small biopsies to be used to test multiple therapies in parallel (either using live micro-fragments or single-cell digested samples), on a scale not currently possible with macro-size tools. The resulting increased throughput of information extracted from small biopsy samples means that more samples can be tested and more quickly, without the need for long periods of cell expansion from biopsy cultures. This increases the feasibility of producing results in a clinically meaningful time-frame for personalised therapy. Moreover, the use of human clinical samples is much more likely to identify clinically relevant mechanisms of resistance than immortalised cell lines or animal models.^97^ Together with increased use of human tissue, there will be a requirement for non-animal derived hydrogels and supplements to be developed and used, as cross-species contamination could adversely affect the predictive value of the assays.

It is expected that over the next few years, the ability of microfluidic assays to predict clinical outcomes using patient samples will be assessed initially retrospectively and then prospectively. This will drive confidence in the technology and facilitate regulatory approval when using *in vitro* models. Increasing success in the development and validation of new microfluidic I/O assays is expected to drive the integration of the technology first into routine R&D practise in the pharmaceutical industry and, subsequently, into clinical care for precision medicine.

However, limiting factors are currently present that do not facilitate access to patient samples and collection of clinical information. New policies should be put in place to accelerate and simplify access to and sharing of patient samples and clinical/health data for research at the national and international levels. This is a key aspect to the faster development of the technology and its global use in the biotech industry. Longer term, the use of functional assays with patient-derived tissue could be a game changer in selecting effective personalised therapies for patients.

In the past 10 years, there has been an explosion of biotechs and Pharma investing in new programmes for developing autologous and allogeneic cell therapies (*i.e.* Dendreon, Kite, Novartis, Aivita, Iovance Biotherapeutics, Leucid, Adaptimmune, Immunocore, Carisma and Allogene to name a few). However, the majority of associated *in vitro* tests are carried out in 2D or using standard well plate technologies prior to animal models. The miniaturization capabilities combined with the large data throughput of microfluidic systems offer a unique opportunity to integrate human tissue-based advanced assays into industrial preclinical studies for the development of stratified therapeutic approaches. Since microfluidics can be used for large-throughput screening, existing immunotherapies could be tested in combination with novel agents as part of a combinatorial regime.^29,98^ The latter is expected to become a particular area of interest as the effect of cell therapy can be enhanced when applied in combination with chemotherapies, radiotherapies or ICIs.^99^

The landscape depicted in this review shows that in the field of immunotherapy for solid tumours, the use of the technology is maturing, but is still in the early stages. A coordinated effort from a variety of disciplines is required to widen the field of application, to produce clinically relevant data and to promote constructive engagement between developers and end users of new microfluidic technologies.

## Conflicts of interest

There are no conflicts to declare.
